# Data From a Mindfulness Program for Family Caregivers to Persons With DD, and Application for Older Individuals

**DOI:** 10.1093/geroni/igaa057.1640

**Published:** 2020-12-16

**Authors:** Jeffrey Kahana, Lawrence Force, Roger Monthie

**Affiliations:** Mount Saint Mary College, Newburgh, New York, United States

## Abstract

Parents who care for their children (young and adult) with developmental disabilities face many stressors and challenges. This paper reports on an intervention using mindfulness and cognitive reframing to improve psychological well-being of care-givers. We report on results based on 92 care-givers who participated in the program. An innovative component was utilizing parent care-givers along with trained peer facilitators. The program was conducted over six weeks, with three in person sessions, and three at home web-based sessions. The content emphasized mindfulness practice (meditation) along with cognitive reframing (aimed at boosting optimism) to address the stress family-caregivers experience in managing worry and the perceived lack of control that accompanies caring for children (young and adult) with developmental disabilities. Post-test data revealed increased awareness of stress coupled with greater competence in stress management. Given the life-long demands for care-giving of parents to the developmentally disabled, normative stressors of aging interact with stressors posed by care-giving demands. Implications for improving well-being of older parental care-givers will be discussed around the topics of (1) optimism and hope; (2) support of healthy behaviors; and (3) development of a mindset of gratitude.

